# Effects of Abiotic Factors on HIPV-Mediated Interactions between Plants and Parasitoids

**DOI:** 10.1155/2015/342982

**Published:** 2015-12-15

**Authors:** Christine Becker, Nicolas Desneux, Lucie Monticelli, Xavier Fernandez, Thomas Michel, Anne-Violette Lavoir

**Affiliations:** ^1^French National Institute for Agricultural Research (INRA), University of Nice Sophia Antipolis, CNRS, UMR 1355-7254, Institut Sophia Agrobiotech, 06903 Sophia Antipolis, France; ^2^Institut de Chimie de Nice, UMR CNRS 7272, University of Nice Sophia Antipolis, Parc Valrose, 06108 Nice Cedex 2, France

## Abstract

In contrast to constitutively emitted plant volatiles (PV), herbivore-induced plant volatiles (HIPV) are specifically emitted by plants when afflicted with herbivores. HIPV can be perceived by parasitoids and predators which parasitize or prey on the respective herbivores, including parasitic hymenoptera. HIPV act as signals and facilitate host/prey detection. They comprise a blend of compounds: main constituents are terpenoids and “green leaf volatiles.” Constitutive emission of PV is well known to be influenced by abiotic factors like temperature, light intensity, water, and nutrient availability. HIPV share biosynthetic pathways with constitutively emitted PV and might therefore likewise be affected by abiotic conditions. However, the effects of abiotic factors on HIPV-mediated biotic interactions have received only limited attention to date. HIPV being influenced by the plant's growing conditions could have major implications for pest management. Quantitative and qualitative changes in HIPV blends may improve or impair biocontrol. Enhanced emission of HIPV may attract a larger number of natural enemies. Reduced emission rates or altered compositions, however, may render blends imperceptible to parasitoides and predators. Predicting the outcome of these changes is highly important for food production and for ecosystems affected by global climate change.

## 1. Introduction

Plants emit volatile organic compounds in considerable amounts: each day, land plants release up to 10% of the carbon they assimilated from carbon dioxide (CO_2_) back into the air [1]. The blend of plant volatiles (PV) emitted by leaves comprises a diverse array of compounds (Table 1), mainly terpenoids as well as fatty acid derivatives, benzenoids, phenylpropanoids, and other amino acid derivatives, methanol and ethylene [2, 3]. While some are emitted constitutively, herbivore feeding and oviposition lead to the release of a special blend of PV called herbivore-induced plant volatiles (HIPV; [4]) and oviposition-induced plant volatiles (OIPV; [5]), respectively. HIPV and OIPV comprise compounds of the same classes that are either not produced by undamaged plants or emitted in different amounts by damaged ones [4]. HIPV have a high information content which is coded by quality and quantity of the HIPV blend [4]. Presence and concentration of, as well as ratio between, compounds can convey highly specific information on the involved herbivore and plant species, possibly even giving away their developmental stages and the plant cultivar [6–9].

As multifunctional infochemicals, HIPV constitute a subcategory of semiochemicals which are mediating interactions between plants and several trophic levels of insects [10, 11]. On the one hand, HIPV can act as allomones in direct defence, having toxic and/or repellent effects on herbivores [8, 12]. On the other hand, they can be perceived by natural enemies (parasitoids/predators) of herbivores who use them to detect their host/prey [13]. This fascinating interaction is also called “cry for help” [4, 14]. Here, they function as synomones—beneficial for both participating parties—providing indirect plant defence by attracting natural enemies of the herbivores and facilitating host/prey detection for the latter [4, 15].

Being sessile organisms, plants cannot run away from pestering herbivores. Chemical communication to summon their enemy's enemy is an important element of their armoury. This kind of indirect plant defence appears to be rather common: it has been described for 49 plant species from 25 different families and insects from 5 different orders [16]. Tritrophic interactions between plants, herbivores, and their natural enemies are presumed to have a long history of coevolution [17]. Recently, using genetically modified tobacco (*Nicotiana attenuata*), the ability of HIPV to increase plant fitness has been demonstrated in field assays for the first time [18].

HIPV can be emitted from bursting storage organs like resin ducts, glandular trichomes, and vacuoles or can be synthesized* de novo* in the damaged tissue or nearby [3]. Triggers for HIPV emission are of chemical as well as physical nature. Mechanical damage to plant foliage alone, imitating herbivore feeding behavior, can result in PV emissions similar but not identical to HIPV [5, 13, 19]. Additionally, specific molecules in the oral secretions of herbivores and the high pH of the secretions elicit the release of HIPV [2]. The mechanisms of how plants “sense” that they are under attack from herbivorous arthropods have been reviewed in detail by Hilker and Meiners [5]. In general, chewing and piercing/sucking insect herbivores are sensed differently and trigger different defence pathways. Chewing insects cause extensive tissue damage. Fatty acids, like linoleic acid which is originally incorporated in cell membranes, are degraded and transformed into C6 and C9 aldehydes, alcohols, ketones, and their esters which are also called “green leaf volatiles” (GLV) due to their characteristic smell [20]. Together with salivary secretions, this induces a defence pathway in which jasmonic acid plays a major role and leads to the emission of specific HIPV as well as the production of specific defence compounds like proteinase inhibitors [8]. Piercing/sucking insects cause way less mechanical damage and are therefore mainly sensed via elicitor molecules from salivary secretions. They activate a defence pathway in which salicylic acid plays a major role, leading to systemic acquired resistance and HIPV production. Regarding this insect feeding guild, relatively little is known about the involved pathways [8]. Both the jasmonic and the salicylic acid dependent signalling pathways lead to HIPV emission. Several biosynthetic pathways are involved in the production of plant volatiles. The main ones are the mevalonate (MVA) and methylerythritol phosphate (MEP) pathways which are producing terpenoids and carotenoid derivatives, the shikimate and phenylpropanoids pathway producing benzenoids and phenylpropanoids, and the lipoxygenase (LOX) pathway producing GLV, methyl jasmonate, and other fatty acid derivatives [20]. All of them have been previously reviewed and described in detail [16, 20, 21]. Therefore we will only give a brief summary (Table 1) and refer the interested reader to the mentioned publications.

The “cry for help” is a very specific interaction phenomenon. Parasitoids are only attracted by volatile blends which correspond to their host. This information is not necessarily conveyed by major compounds in the blend; minor compounds or compound ratios can be just as important [4]. In the context of current global change, the question arises if HIPV are affected by changing abiotic conditions. Just as plants cannot run away from herbivores, they cannot escape unfavorable changes regarding climate, atmosphere, or soil by going somewhere else. In order to survive, they have to acclimate or adapt. Hence, abiotic factors have a strong impact on plant metabolism. Typically, organic compounds produced by plants are grouped into “primary” and “secondary” metabolism. Primary metabolism includes the very fundamental compounds which mainly consist of fatty acids, amino acids, and sugars. Secondary metabolism comprises compounds which are not imperatively necessary for the plant's survival but can be extremely beneficial and often are of high ecological value, like chlorophyll, flavonoids, alkaloids, terpenoids, and many more.

Abiotic factors affecting primary metabolism are particularly well known.

*Climate*. Temperature directly affects enzymatic activity and kinetics of chemical reactions. The intensity of photosynthetically active radiation (PAR) strongly influences the photosynthetic rate and stomatal conductance and, thus, the availability of carbohydrates as precursors for a plethora of biosynthetic pathways. Ultraviolet (UV) radiation is not used for photosynthesis but is very energetic and can lead to mutations by causing pyrimidine dimers in the DNA. Air humidity also affects stomatal conductance and thereby the plants evapotranspiration and xylem flow which transport nutrients from the roots to the leaves. 

*Atmosphere*. The CO_2_ concentration strongly affects the carbohydrate pool of plants by supplying carbon, influencing photosynthesis, and stomatal conductance. Ozone (O_3_) on the other hand is highly reactive and can expose plants to oxidative stress due to enhanced formation of reactive oxygen species (ROS). 

*Soil*. The availability of water is extremely important for plants. Like other factors, it affects photosynthesis and stomatal conductance as well as many more processes in cells. Availability of nutrients in the substrate the plant dug their roots into is another essential factor. Nitrogen, for example, is incorporated in molecules as fundamental as DNA, RNA, proteins, and chlorophyll. Maintaining their mineral homeostasis can be a challenge for plants: they require a certain level for signalling cascades, osmotically regulated processes, and as enzyme cofactors but high levels can be toxic. High salt concentrations can, for example, cause hydric deficit and osmotic shock. 

Abiotic changes mostly do not affect one single process but rather a whole range. Primary and secondary metabolism are not two strictly separated pathways but interact and are intertwined in many ways, production and consumption of carbohydrates being only one of these intersections. Because of these interconnections, abiotic factors impacting primary metabolism are likely to have consequences for secondary metabolism. Plant volatiles are classified as secondary metabolites. Considering their involvement in plant defence, the question arises if plants are still able to defend themselves directly and indirectly in a changing environment. Contrary to constitutive PV emission, the effects of abiotic factors on HIPV-mediated biotic interactions have received only limited attention to date [22]. If the emission of constitutive PV is affected by changing abiotic factors, is this also true for HIPV? Are their quantity and quality consistent although the plant's growing conditions change? Studying the impact of abiotic factors on constitutive plant volatiles provides the opportunity to elucidate their potential effect on HIPV since they share the same biosynthetic pathways.

## 2. How Do Abiotic Factors Impact Constitutively Emitted Plant Volatiles?

Constitutively emitted plant volatiles have several ecological and metabolic functions. They can attract pollinators and seed dispersers, act as direct defence against herbivores and pathogens, and mediate plant-plant-signalling as well as protecting plants against high temperatures, high light intensity, and oxidative stress [1, 3, 23]. Abiotic factors generally affect the emission of PV which has been discussed and summarized in several reviews [3, 24]. Hence, in the following, we will only provide a brief overview. 

*Climate*. High temperature impact has been studied in short and long term experiments [24]. Temperature immediately affects the vapour pressure of compounds, stomatal aperture, enzymatic activity, and availability of precursor molecules [25]. Consistent with accelerated kinetics of biochemical reactions (Q10 rule), DeLucia et al. [26] found increased concentrations of defensive compounds related to both jasmonic acid and salicylic acid signalling pathways in several plants. In the long run, physiological acclimation and altered gene expression patterns additionally play a role [24]. Although long term studies are scarce, Peñuelas and Staudt [24] refer to rising temperatures as increasing constitutive isoprenoid emission in short and long term experiments. Increased isoprene emissions have been linked to enhanced thermotolerance due to improved lipid membrane stability [27, 28]. However, there are counterexamples where PV emission is not affected by temperature or is decreasing [24, 29]. Niinemets et al. [25] emphasize that the emission of many PV is strongly light-dependent. Among other effects, the intensity of PAR affects stomatal aperture and photosynthesis rates which in turn affects the availability of carbon-based precursor molecules for biosynthesis [25]. In agreement, elevated intensity of PAR has been observed to increase photosynthesis and isoprene emissions in two tropical tree species [30]. Furthermore, terpenoids can function as photoprotectants by dissipating energy and/or scavenging ROS in photosynthetic membranes [31]. Consistently, enhanced exposure to radiation from the UVB spectrum is also reported to increase emission rates of constitutive PV although there appears to be considerable variation depending on the studied species and the applied doses [24]. The “opportunist hypothesis,” however, suggests that terpenoid emission is a byproduct of the biosynthesis of essential isoprene-based compounds like carotenoids [32]. The authors propose that emission of terpenoids is high under conditions leading to accumulation of essential isoprenoids, solely because they have common precursors and terpenoids (up to C15) are very volatile. On the other hand, Vickers et al. [33] suggested that isoprenoids generally improve the plant's tolerance to internal oxidative stress regardless which external factor caused it. 

*Atmosphere*. The effect of elevated CO_2_ concentrations was unclear in long and short term experiments reviewed by Peñuelas and Staudt [24], although a large number of studies report decreasing PV emissions. DeLucia et al. [26] confirm the considerable variance in the effect of CO_2_ concentration on PV emissions, especially when comparing different species. Still, the authors detected the general trend in the literature that elevated CO_2_ stimulates the production of phenolics in general but especially tannins and flavonoids while suppressing the production of terpenes. They summarize that the shikimic pathway, regulated by salicylic acid, appears to be enhanced in high CO_2_ concentrations while the MEV and MVA pathways, regulated by ethylene and jasmonic acid, appear to be repressed. Peñuelas and Staudt [24] reviewed many indications of increasing emission of constitutive isoprenoids due to enhanced ozone exposure. Ozone poses an oxidative threat and can therefore increase the biosyntheses of antioxidants, like isoprene [33, 34], and, furthermore, have an additional effect on PV by degrading molecules once they have been emitted from the plant [24, 34, 35]. 

*Soil.* At first glance, the literature is ambiguous regarding the effect of drought. Looking closer, however, the issue resolves into a dose-dependent response with some variance due to plant species, drought duration, and method used to measure drought: mild drought may increase emissions or have no effect [24] but severe drought generally decreases emissions [36]. Still, approaching the complex situation using a model, the majority of variation in plant isoprene and monoterpene emission could be explained by variation in temperature and light, as well as leaf area index and plant functionality [37, 38]. In Mediterranean ecosystems where drought periods are typical climate events, temperature and PAR are not enough to simulate monoterpene emissions: adding a module on soil water content is necessary to improve simulations [39]. The impact of soil could be strongly species-dependent: terpene emissions of* Rosmarinus officinalis* and* Pinus halepensis* were generally higher on calcareous than on siliceous soil while it was the other way around regarding* Cistus albidus* [40]. The same pattern emerged regarding* Cistus monspeliensis*: terpene emissions were 7 times higher on siliceous than on calcareous substrate [41]. Increasing N-supply has been found to increase isoprenoid emissions [24]. High phosphorous supply, however, coincided with low isoprene emissions in* Phragmites australis* [42]. Salt stress had no effect on isoprene emissions of* Eucalyptus globulus* and* Populus x canescens* [43, 44]. 

In summary, abiotic factors can affect PV emission at a physiological (e.g., availability of precursors for biosynthesis and enzyme activity) and/or a physicochemical level (e.g., vapour pressure of the compound of interest, the leaf internal structure, and stomatal aperture) [25]. While a lot of single factors have been studied, there are also countless examples of their interactions [24]. Although rising temperature may increase precursor availability and enhance the compounds' vapour pressure, it can also decrease stomatal aperture [24]. The latter would also be the effect of drought [24]. Deficiencies regarding water and/or nutrient supply as well as salt stress may disturb the osmotic status of plants and therefore likewise affect stomatal aperture.

Chemically, constitutively emitted and induced plant volatiles are not always clearly distinguishable. Terpenoids, fatty acid derivatives, benzenoids, and phenylpropanoids are present in both groups [4]. Some may be emitted in higher concentrations or altered ratios after herbivore attack while other compounds are emitted exclusively then [4]. Both constitutive and induced plant volatiles are derived from the same biosynthetic pathways (Table 1). In order to further illustrate the compounds' resemblance regarding their structure, we assembled examples for both categories of HIPV—involving quantitative or qualitative changes (Table 2). It is highly probable that factors which affect the biosynthesis and emission of constitutive PV also affect HIPV because they depend on the same pool of resources and energy.

## 3. Do Abiotic Factors Affect the Emission of HIPV?

Plants only emit induced volatiles in certain situations. According to the Optimal Defence Hypothesis [45], only producing compounds when they are needed saves resources because plant volatiles come with a metabolic cost. For instance, maize plants that were genetically modified to constitutively emit the HIPV (*E*)-*β*-caryophyllene and (*E*)-*α*-humulene showed decreased fitness compared to nonmanipulated plants which only emit these compounds when under attack [46]. However, studies suggest that maintaining signalling pathways may also have considerable metabolic costs for plants [47].

Regarding HIPV emission, there is a large variance caused by several biotic and abiotic factors. Major biotic factors affecting HIPV are the plant species or even the cultivar concerned, the plant organ the damage is afflicted on, the extent of the damage and its duration, and both the feeding guild and species of the herbivore as well as the ontogenetic stage of both plant and herbivore [13, 22, 48]. During plant ontogeny, quantity and quality of HIPV emission change with higher concentrations emitted during their vegetative compared to their reproductive stage [9]. Major abiotic factors affecting HIPV are temperature, light intensity, and ozone, as well as water and nutrient availability [3, 16, 49]. The influence of abiotic factors on HIPV has received much less attention than that on constitutive PV [22]. Generally, all factors that influence stomatal aperture could affect HIPV emission [50]. However, there is heterogeneity in the literature and results are not consistent [3]. The most detailed study on the effect of abiotic factors on HIPV has been published by Gouinguené and Turlings [29] who applied* Spodoptera littoralis* regurgitant on mechanically wounded leaves of young maize plants. Their results are listed in the following paragraphs. Except for air humidity, all tested abiotic factors caused qualitative changes of the HIPV blend. Interestingly, they detected considerable differences among the studied compounds. 

*Climate*. Elevated temperature appears to enhance jasmonic acid, salicylic acid, and ethylene synthesis [26], all of which are involved in plant defence. Yet, the effect of temperature strongly depends on the temperature optimum of the respective plant species [26]. In young maize plants treated with* S. littoralis* regurgitant, HIPV emissions were highest between 22 and 27°C which probably correlates with maximum stomatal aperture [29]. At a relative air humidity of 60%, HIPV emissions were highest—compared to higher or lower humidity—in young maize plants which may also be explained by high stomatal aperture [29]. The authors also report that HIPV emission was generally light-dependent, increasing with radiation intensity, and did not happen in the dark. They suggest that HIPV production is closely connected to photosynthetic activity. Herbivores with strong diurnal feeding rhythms may also add diurnal variation to HIPV emissions: less feeding activity at night is common with many insect herbivores and some HIPV emissions are closely related to herbivore pressure [51]. Still, there is a considerable variation of response and/or emission patterns among HIPV: in cotton plants infested with* Spodoptera exigua*, the induced terpenoids (*E*)-*β*-ocimene and (*E*)-*β*-farnesene were emitted in a pronounced diurnal pattern while *α*-pinene and caryophyllene were not [52]. Additionally, (*E*)-*β*-ocimene emission continued to follow the diurnal pattern even after the caterpillars had been removed. In contrast, *α*-pinene emission stopped after insect removal. The authors hypothesize that the emission patterns especially of HIPV released from damaged storage organs depend on the feeding rhythm of the herbivores [52]. Although UVB radiation is known to activate the salicylic acid, jasmonic acid, and ethylene pathways, little is known about its potential to change HIPV [16]. Considering that enhanced UVB radiation can increase isoprene emission in some species [53], it is possible that it also affects HIPV. 

*Atmosphere*. Elevated CO_2_ concentrations affect the salicylic and jasmonic acid dependent defence pathways differently [8]. Studies suggest that elevated CO_2_ concentration suppresses jasmonic acid while stimulating the production of salicylic acid [26] which may improve the plants ability to sense and signal attacks of piercing/sucking insects but hamper signalling pathway related to chewing insects and the respective production of HIPV. Elevated O_3_ levels reduced the total terpenoid emission of nontransgenic and transgenic Bt-*Brassica napus*—oilseed rape which is producing insecticidal* Bacillus thuringiensis* toxin [35]. As mentioned in the previous section, O_3_ enhances the level of oxidative stress in plants [16]. Some terpenoid volatiles have antioxidant activity and thus can ameliorate the oxidative damage—they may be synthesized by plants to scavenge ROS [16, 17]. The elucidation of causes and consequences of changed HIPV in elevated O_3_ levels is further complicated by its high reactivity which can lead to secondary changes of already emitted HIPV [24, 35]. 

*Soil*. The salicylic and jasmonic acid dependent defence pathways are also differently affected by drought stress [8]. HIPV emissions of young maize plants were higher in dry compared to wet soil, possibly because water stressed plants invest more in the biosynthesis of defence compounds [29]. In* B. napus* plants grown on nutrient deficient soil, the emission of several HIPV decreased compared to well-nourished ones [54], while HIPV emission of tobacco was not affected by low soil nitrogen [55]. Nitrogen-starved soy plants (*Glycine max*) produced the same range of HIPV like the well-nourished plants, but three compounds were affected in their concentrations [56]. Varying severity of nutrient deficiency may be a crucial factor to explain this heterogeneity of results. Salinity can alter the composition of HIPV blends in maize plants and reduce emissions per plant because it reduces plant growth [57]. Heavy metal stress also has the potential to affect volatile emissions. However, while maize plants exposed to copper stress emitted higher levels of HIPV when damaged by* S. frugiperda*, cadmium-exposure did not result in differential emissions [58]. 

These examples illustrate the plant side of the interaction, focussing on production and/or emission of induced compounds. In their review, Peñuelas and Staudt [24] pose the question whether the defensive function of HIPV will be retained if their quality or quantity is affected by abiotic changes. Will the parasitoids still be able to decipher the airborne message or will they be confused by the changes? In the following section we will focus on the parasitoid side of the interaction.

## 4. Do Abiotic Factors Impact Higher Trophic Levels through HIPV?

The emission of HIPV is a well-known characteristic interaction “cry for help” between the first and third trophic levels: the plant and the parasitoid. The chemical composition of a plant, that is, its nutritional value as well as concentrations of chemical defence compounds, shapes the arthropod community that interacts with the plant, notably with respect to the community's size, density, and dynamics [59]. On nitrogen-deficient plants, for instance, herbivore survival can decrease through bottom-up effects [49]. Increased C/N ratios make it harder for herbivores to cover their own nitrogen demand and this can prolong feeding time and slow down their development. This may increase the probability of parasitoids detecting and parasitizing the herbivores and the host may stay in vulnerable stages for a longer time [60]. However, the hosts themselves may be of lower nutritional value for the parasitoids larvae and/or may contain higher concentrations of plant defensive compounds, potentially toxic toward parasitoid larvae [59].

Even if parasitic hymenoptera do not directly feed on plant tissue, they can be affected through bottom-up effects of the plant's nutritious value and/or secondary metabolites which can promote or impede the plants “cry for help” [59]. Abiotic factors which alter the quality or quantity of HIPV may render the chemical “message” incomprehensible to the receiving organisms. Increased HIPV emission due to optimized temperature or air humidity as well as increased PAR intensity may improve signal perception for the parasitoid, also in greater distance of the emitting plant. Ozone, with its high reactivity, could decompose the volatiles and, thus, eliminate the signal. Drought and nutrient deficiency can decrease HIPV concentrations which may make it impossible for parasitoids to locate the plant or to even perceive the signal in the first place, if they are not in the direct vicinity.

One approach to predict if altered HIPV blends will affect parasitoids is to elucidate which compounds they can perceive. Coupled with GC-MS, the electroantennographic detector (EAD) allows for separation and identification of compounds and therefore provide a screening method for compounds which may be behaviorally active [16]. Gouinguené et al. [61] observed via GC-EAD that three species of parasitic hymenoptera,* Cotesia marginiventris*,* Microplitis rufiventris*, and* Campoletis sonorensis*, are able to perceive a variety of HIPV induced by* Spodoptera littoralis* larvae feeding on maize, cowpea, or cotton plants, some of which were only minor compounds. However, whether perception actually leads to a behavioral response and whether it will be positive or negative can only be investigated in observational studies [16]. It is hardly possible to predict the effect a changed blend of HIPV will have on parasitoids. As illustrated by the following examples, changed blends do not necessarily affect the natural enemy's behavior. 

*Climate*. To our knowledge, the effects of temperature and PAR have not been tested yet. Exposure to UVB radiation did reduce oviposition and larval feeding of the moth* Plutella xylostella* on two Brassicaceae species, while increasing parasitization by* Cotesia plutellae* [62, 63]. However, we do not know which are the underlying mechanisms. Caputo et al. [62] report an example of parasitoids discriminating between hosts feeding on UV- and non-UV-exposed plants. While this may be explained by altered HIPV composition, it may just as well be due to other factors like changed host quality. On the other hand,* Cotesia marginiventris* did not discriminate between* Spodoptera frugiperda* larvae feeding on UV- or non-UV-exposed soybean (*Glycine max*) plants [64]. 

*Atmosphere*. Elevated CO_2_ concentrations affect the feeding guilds differently: while phloem feeders tend to respond positively to the elevated carbohydrate level in plants, foliage feeders tend to respond negatively—possibly due to lower nitrogen concentrations (higher C/N ratio) or due to increased defence compounds [8]. This may in turn affect their parasitization rates. Minor changes of HIPV due to elevated CO_2_ concentrations can jeopardize the interaction between parasitic wasps of the genus* Cotesia* and moth-infested (*P. xylostella*)* Brassica* species [65] or not [35]. It is unclear whether this is due to the different species of* Brassica* and* Cotesia* studied. While elevated O_3_ levels reduced the total terpenoid emission of nontransgenic and transgenic Bt-*B. napus*, only the latter was negatively affected in its ability to attract* Cotesia vestalis* [35]. In a different study, however, the communication between* P. xylostella*-infested* Brassica oleracea* and* C. plutellae* was not disrupted, even though elevated O_3_ concentrations completely degraded most herbivore-induced terpenes and GLV [66]. The authors suggest that the successful orientation of the natural enemies may have been due to less reactive HIPV like benzyl cyanide and methyl salicylate, respectively. However, as completely clean air is rare in nature, Holopainen et al. [17] suspect that parasitoids may have learned to also associate the breakdown products of HIPV with their host and possibly even use the ratio between originally emitted compounds and their reaction products to estimate the plant's distance. Increased isoprene concentration in the plant periphery of genetically manipulated* A. thaliana* repelled the parasitic wasp* Diadegma semiclausum* but not* Cotesia rubecula* or the lepidopteran herbivores* Pieris rapae* and* Plutella xylostella* [67]. 

*Soil*. Nitrogen deficiency has strong bottom-up effects on the leaf miner* Tuta absoluta* feeding on tomato leaves [49], as well as on* S. frugiperda*, feeding on nitrogen-deficient soybean leaves, and its parasitoid* C. marginiventris* [56]. However, the latter authors concluded that indirect plant defence was not compromised because the behavioral response of the parasitoid to the emitted HIPV was unchanged. In maize seedlings treated with the elicitor volicitin, sesquiterpene emissions were higher in nitrogen-deficient compared to nondeficient plants [68], which may improve the attraction of parasitoids. 

Additionally, plants may offer shelter or nectar as a food source for parasitoids [59] which may in turn be affected by abiotic conditions: Adler et al. [69] found higher concentrations of alkaloids in nectar of* Nicotiana tabacum* plants that were well fertilized compared to those receiving less nutrients. Such increased concentrations of toxic compounds in nectar may have direct negative effects on survival and/or fitness of parasitoids.

HIPV furthermore have the potential to mitigate various additional interactions among other organisms present in the community. Sensing the presence of beneficial or detrimental organisms, plants can change chemically and/or morphologically with effects on the arthropod communities. Plant defensive compounds can affect the community composition by repelling generalist herbivores but serving as recognition cues to specialists who can detoxify or sequester them for their own defence (e.g., see Desneux et al. [70]) and, thus, indirectly affect the composition of higher trophic levels [59]. HIPV can also have adverse effects on the emitting plants: they may come with negative ecological costs like repelling pollinators [71–73]. Furthermore, communication by volatile compounds is not necessarily a secure connection encrypted to outsiders. Other receivers may be “eavesdropping” on the plant-emitted signals and exploit the intercepted information. Some herbivores use HIPV to find suitable host plants [74] which can turn HIPV into plant kairomones—disadvantageous for the plants themselves. Communication also happens inside the plant community: not-infested neighbouring plants can perceive HIPV and boost their own defence without having suffered from herbivory themselves [15]. Eventually, it seems logical that a system has to be complex to convey information as detailed as observed regarding plant-insect communication. Yoneya and Miki [11] suggest that the multifunctionality of HIPV and variations in plant responses to herbivory are key mechanisms for evolutionary diversification of animal foraging and therefore the structure of ecological networks. Kessler [47] argues in the same direction, suggesting that the dynamics of induced defence compounds, like HIPV, with all the inherent complexity and multifunctionality, should also be seen as an information network.

These studies illustrate how plants are influenced by their environment and how these changes can be propagated through the higher trophic levels as bottom-up effects. Climate, soil, and atmosphere have a large potential to impact parasitoids directly or indirectly through plants. As they are often employed as biological pest control agents, a dramatic question arises: will global change jeopardize integrated pest management? Can we confront this looming threat with detailed knowledge on the elements involved? Moreover, could we go one step further and even use this knowledge to our advantage and improve the efficacy of parasitoids by manipulating the plants' growing conditions?

## 5. Significance of HIPV for Integrated Pest Management and Future Prospects

### 5.1. Could Optimized Abiotic Factors Improve Integrated Pest Management?

The effects of abiotic conditions on HIPV and/or on natural enemies have mostly been studied focussing on factors relevant in a changing global environment [16, 24, 75]. However, some of these factors are also relevant in horticultural and agricultural context where parasitoids are often employed as pest control agents. Temperature, water supply, and humidity, for instance, are likewise affected by climate change and managed in horticulture and, to some extent, in agriculture. Plant nutrition, irrigation, temperature, and radiation intensity as well as CO_2_ concentration are closely controlled in many modern horticultural production systems. In agricultural production systems, fertilization and irrigation are often manipulated. This may offer opportunities to adapt cultivation practice during biological pest control application to maximize the natural enemies' performances. 

*Climate*. Crop producers using greenhouses may be well advised to increase heating and decrease cooling, respectively, decrease the application of shading screens or add lamps, and adjust a relative humidity of 60% to increase HIPV emission. However, we have to bear in mind that, so far, there is a lack of studies regarding the response of the parasitoids. Furthermore, one can assume that the optimal values for temperature, radiation intensity, and relative air humidity are immensely dependent on both the involved plant and insect species. It is well possible that optimal climatic conditions for high crop yield are not the same as for good parasitoid performance. High temperature, for example, might have positive effects on HIPV emission but is prone to have negative effects on yield. Regarding many tritrophic systems, finding compromises might be challenging. 

*Atmosphere*. CO_2_ enrichment is a common practice in greenhouse crop production. A meta-analysis found that elevated CO_2_ concentrations decrease herbivore abundance but increased foliage consumption [60]. The “high carb diet” slowed down herbivore development and, hence, may lead to increased attack rates by natural enemies because of higher exposure time [60]. This may be beneficial when parasitoids are employed to control foliage feeders. However, each tritrophic system has to be evaluated carefully as the response of parasitoids to bottom-up changes due to elevated CO_2_ concentration has been observed to vary substantially (see Section 4). 

*Soil*. In hydroponic cultivation systems, nutrient and water supply can be easily manipulated. Reducing the amount of nutrient solution or the frequency of its supply may increase HIPV emission in some plants by establishing mild drought and nutrient deficiency. However, greenhouse crops have hardly been studied in this respect. As we illustrated in the previous sections, existing results are promising but also highlight the interspecific variability. 

While existing results definitely show tendencies, clearly more research is needed [24]. Attention has furthermore to be paid to temporal changes in HIPV blends which can affect herbivore and parasitoid preferences [76]. Additionally, most studies investigated the effect(s) of one altered factor while in a changing global context several factors will interact which calls for studies on various factors simultaneously [17].

### 5.2. Application of Synthetic Blends of HIPV

Instead of manipulating the plant's HIPV emission and to overcome variability due to a variable environment, compounds can be artificially applied to crop production systems to attract natural enemies [77]. Kaplan [78] has recently published a thorough review on HIPV application in biological control, explaining methods and mechanisms, listing which compounds attract which species (target and nontarget effects), describing opportunities and limitations, and we would like to refer the interested reader to his article for further details.

The potential of single synthetic HIPV or of blends in horticulture and agriculture has been the subject of several studies. Simpson et al. [15] list a number of chemicals that were successfully attracting parasitoids in field trials. Namely, these are methyl salicylate,* cis*-3-hexenyl acetate, geraniol, methyl anthranilate, methyl jasmonate,* cis*-jasmone,* cis*-3-hexen-1-ol, 3,7-dimethyl-1,3,6-octatriene, farnesene, octyl aldehyde, and indole. Topic application of plant hormones like jasmonic or salicylic acid can lead to the release of PV, but the quality and quantity mostly differ from actual HIPV blends [79]. In a large study, comparing the effect of methyl salicylate,* cis*-3-hexen-1-ol, and phenylethyl alcohol in maize and soybean fields, 4 and 16, respectively, out of 119 arthropod taxa showed significant responses [80]. The authors summarize that, all in all, repellent effects of HIPV were as frequent as attractive effects and the crop studied has a strong influence. Gols et al. [81] made the appeal that, in order to gain a more realistic understanding of these interactions in an ecological and evolutionary framework, studies should not simply focus on crops but involve wild plants. They found* C. rubecula* to be more attracted to wild than to cultivated cabbage infested by* P. rapae*.

Synergistic effects of blends of HIPV compared to single compounds have been observed a number of times regarding attraction of parasitic wasps [80]. While 13 synthetic HIPV showed activity in EAGs of* Cotesia sesamiae*, only 3 of them elicited behavioral responses when tested at a natural dose and two more at a higher dose [82]. Still, the authors had to combine 9 compounds to create a synthetic HIPV blend that was as attractive as the natural blend emitted by maize plants infested with female stemborers (*Chilo partellus*). Consistently, a study on genetically altered* A. thaliana* found a blend of HIPV and constitutively emitted PV to be more attractive to* C. marginiventris* than the HIPV alone [83]. Studies on natural enemies different from parasitoids point in a similar direction [84, 85]. An explanation of these synergistic effects might be that more compounds can convey more, thus more specific, information than single compounds. Fontana et al. [83] suggested that a successful host finding strategy might involve both constitutive and herbivore-induced volatiles. Specific information about the involved plant and herbivore species may be especially important for specialist parasitoids (see next section).

The use of synthetic HIPV for pest control in agroecosystems is not without risk. A field study showed that application of one single HIPV common in soybean managed to repel and/or attract several arthropod species in a range of up to 8 m from the source [86]. However, the authors observed that braconids were lured from surrounding fields, resulting in a depletion of braconid communities in neighbouring fields—possibly increasing the risk of herbivore outbreaks there. Removing parasitoids from surrounding areas may furthermore disrupt their population dynamics [87]. Meiners and Peri [87] caution that parasitoids which were artificially attracted to a field with low host density might decrease their foraging rates because the cue does not deliver a reward, that is, available hosts, and that higher parasitoid densities may not necessarily lead to higher parasitization rates. Using synthetic HIPV in the field may furthermore have unwanted effects like attracting additional herbivores and disrupting trophic cascades [88].

### 5.3. Is the Parasitoid's Host Range Relevant to Their Future Employment as Biological Pest Control Agents in a Changing Environment?

Considering the plethora of HIPV compounds, blends, and their variability, the ability of predators and parasitoids to discriminate between the chemical cues is immense [16]. Yet, not all parasitoids necessarily use the same molecules for orientation.

As illustrated in the previous section, there is a considerable heterogeneity among the observed responses of parasitic hymenoptera to plant volatiles. With two studies, Ngumbi et al. provided some more detailed insights about the complexity of parasitoid responses, suggesting that the sex of the insect has an influence, as well as its degree of host specialization. In Y-olfactometer essays, females of* Cotesia marginiventris* and* Microplitis croceipes*, a generalist and specialist parasitic hymenoptera species, responded stronger to HIPV than their respective males [89]. Additionally, at low dosages, the authors observed the generalist to respond strongly to GLV which convey the general information that herbivory is taking place while specialists responded stronger to more specific, host-related HIPV. These behavioral essays correspond well to earlier GC-EAD studies [90, 91]. The authors suggested that specialists use differences regarding compound ratios to determine if the feeding insect is their host or not. Specialists are considered to be rather “narrowly tuned” on host-related volatiles while it is sufficient for generalists to register broad-spectrum herbivory cues like GLV [10, 90–93]. Again, however, we must be alert to exceptions from this rule. A study comparing the generalist* Diadegma fenestrale* and the specialist* Diadegma semiclausum* did not find behavioral differences [71]. Yet, the authors emphasize the importance of ontogeny: both species only differentiated host and nonhost HIPV produced by the plant species they were reared on.

Associative learning describes a process where responses to certain stimuli are newly acquired or existing responses are enhanced by linking them to a reinforcing stimulus [10]. It has often been suggested to be the mechanism responsible for olfactory learning in adult parasitic wasps, increasing the phenotypic plasticity of displayed responses [10, 94–96]. For example, both* Cotesia glomerata* and* C. rubecula* (parasitoids of first-instar* Pieris brassicae* and/or* P. rapae* larvae) showed increasing interest in a previously unattractive host plant after finding suitable caterpillars there [94]. The author emphasizes that the two wasp species showed substantial differences although they are closely related:* C. glomerata* changed its innate preferences from cabbage odours towards odours of another plant already after one single experience and remembered it for at least five days.* C. rubecula* kept preferring cabbage odour and completely quit responding to the new odour after one day. The authors related the differences in learning to the wasps' social and oviposition behavior as well as the oviposition behavior of their hosts. While both* Cotesia* species are considered specialists,* C. glomerata* is described as more of a generalist than* C. rubecula* [94]. This is mirrored by inconsistent reports in the literature, describing* C. glomerata* either as specialist regarding its insect hosts [97] or as generalist [98]. Lately, another study on associative learning in parasitoids found a greater effect regarding the species with a wider compared to a more specialized host range [99]. While both generalists and specialists use infochemicals to find hosts and both have innate odour preferences, learning capacity is more pronounced regarding generalist natural enemies than specialists [100]. The generalist parasitic wasp* Psyttalia concolor* (Hymenoptera: Braconidae), for example, has been trained to associate the previously unattractive volatiles geranyl acetone, nonanoic acid, and decanoic acid with food rewards and the authors suggested the possibility to train mass-reared wasps before using them as biological control agents [101]. Apparently, innate positive responses to HIPV can also be nullified or even reversed:* P. concolor* trained to associate HIPV with electric shocks, grew indifferent to low concentrations, and avoided high concentrations of the actually attractive HIPV ethyl octanoate and decanal [102].

Parasitoids can be specialists on herbivore and plant level, specialists at plant, and generalist at herbivore level and vice versa, as well as generalists on both levels [10]. In Table 3 we listed several parasitic hymenoptera and their host breadth. The list points out the generalists who, on the one hand, may offer reliable efficacy as pest control agents under variable conditions. Tritrophic systems involving parasitoid species from this category may therefore be less vulnerable to altered HIPV blends induced by varying abiotic conditions. On the other hand, generalists on herbivore but not plant level show the highest potential to be trained in order to increase their efficacy as biological control agents [10]. They could possibly be trained to respond to volatiles which are not subject to changes. Table 3 also points out the specialists. Tritrophic systems involving parasitoids with a narrow host range may, on the one hand, be very vulnerable to changes. This could have major ecologic and economic implications against the background of global change and may be a key concern given how many known parasitoids can be classified as specialist to their host and/or to associated plants. On the other hand, these systems might be optimized by adding crucial compounds or enhancing their biosynthesis in plants. So, to answer the question posed in the subsection's title: yes, there are indications that the parasitoids' response to changing abiotic factors is strongly influenced by their degree of specialization.

Based on the observation that generalists rather tend to respond more to unspecific GLV and specialists to specific, host-related HIPV [89, 100], it would be interesting to know if these groups respond differently to abiotic factors. If one of the compound groups was less susceptible to changes, the respective parasitoid-herbivore-plant system should be more resilient and favorable in unstable environments. Unfortunately, existing data so far do not suffice to draw conclusions. Gouinguené and Turlings [29] do report (*E*)-*β*-farnesene to be emitted in more stable proportions than (*E*)-nerolidol. However, they are sesquiterpenes which are considered rather specific, host-related volatiles. This suggests that the emission is more finely regulated than just based on compound class. Still, this is only one study and in the big picture things might look different. There is a great need to decipher the language used by plants to communicate with insects. Knowing which compounds and/or compound ratios are pivotal for specialists used in biological pest control and how these HIPV are subject to changes due to biotic or abiotic impact factors may be essential for their future employment in food production.

## 6. Conclusion

Abiotic conditions have the capacity to alter the interaction between parasitic hymenoptera and plants. Changes regarding climate, atmosphere, or soil can increase or decrease the emission of constitutive and herbivore-induced plant volatiles. They can have bottom-up effects on parasitoids by affecting their herbivore hosts or influence orientation of parasitoids directly. Some tritrophic interactions are threatened by climate change; others seem more resilient. Active manipulation of abiotic factors in food production systems offers the chance to improve the efficacy of pest control through parasitoids. However, the large variability between the different tritrophic systems and the organisms involved requires thorough investigations and careful application of the gained knowledge.

## Figures and Tables

**Table 1:** Biosynthesis of main compounds classes of herbivore-induced plant volatiles.

*Terpenoids*		
Terpenoids are basically synthesized in three consecutive steps as described by Dudareva et al. [20]: first, formation of the primary C5 units, the isoprene building blocks. Two or more of these C5 units can, in the second step, be condensed into C10 or C15 units which are, in the third step, conversed into the respective mono- or sesquiterpenes. Step two can be skipped to convert a single C5 unit into a hemiterpene. There are two pathways producing the C5 units in plant cells. The MEP pathway is located in the plastids and produces C5 units for hemi-, mono-, and diterpene synthesis. The MVA pathway is located in the cytosol, producing C5 units for sesquiterpene synthesis. Cross talk between these two pathways is happening. Eventually, enzymatic alterations can improve the volatility and/or change functionality of the hemi-, mono-, sesqui-, and diterpenes. The large enzyme family of terpene synthases is responsible for the last steps in terpene biosynthesis, creating an astounding diversity of terpenoids. Volatility decreases with increasing molecule size: hemi- and monoterpenes are considered volatiles while sesquiterpenes are semivolatiles and diterpenes are nonvolatiles.	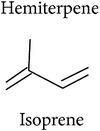	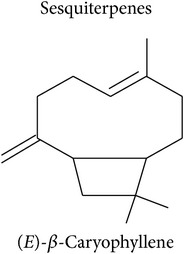
	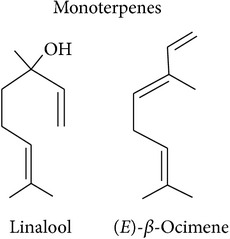	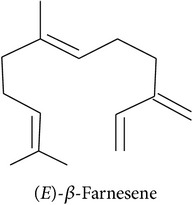

*Benzenoids and phenylpropanoids*		
The shikimate pathway synthesizes the amino acid *L-*phenylalanine which is the common precursor of benzenoids and phenylpropanoids which contribute to the HIPV bouquet [2, 17]. After *L-*phenylalanine is deaminated by the enzyme phenylalanine ammonia-lyase, the resulting trans-cinnamic acid can be transformed into benzoic acid, the precursor of benzenoids, or into phenylpropanol, the precursor of volatile phenylpropenes like eugenol and chavicol. Volatile phenylpropanoids, however, are produced from *L*-phenylalanine directly. Either way, the final biosynthetic steps are dominated by the enzyme superfamilies of acyltransferases and methyltransferases.	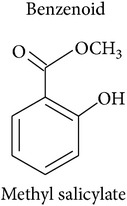	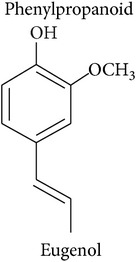

*Fatty acid derivatives*		
The LOX pathway produces derivatives of C18 fatty acids released from damaged cell membranes [20]. Methyl jasmonate and “green leaf volatiles” (GLV) like hexenol and hexenyl acetate are all breakdown products of C18 unsaturated fatty acids like linoleic and linolenic acid [20]. In the first step of the LOX pathway, the fatty acids are stereospecifically oxygenated into 9- or 13-hydroperoxy intermediates, feeding two separate branches of the pathway: methyl jasmonate and C6 GLVs are produced from the 13-hydroperoxy intermediates while C9 GLV are produced from the 9-hydroperoxy intermediates [146].	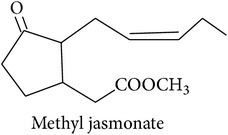	
	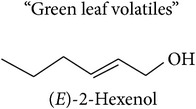	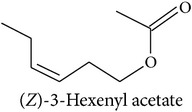

MEP = methylerythritol phosphate pathway, MVA = mevalonate pathway, LOX = lipoxygenase pathway.

**Table 2:** Herbivore-induced plant volatiles emitted constitutively but in increasing concentrations after herbivore attack (quantitative changes) or only after herbivore attack (qualitative changes). Compounds from both categories can have very similar structures and share a biosynthetic pathway (see Table 1). This list is non-exclusive and inter-specific variation can be expected. It is mainly based on results on potato and tobacco (*Solanum tuberosum* and *Nicotiana tabacum*) reported by Dickens [148] and Robert et al. [46] as well as several review articles [16, 20, 147].

Compound class	Constitutively emitted, increasing after herbivore attack	Only emitted after herbivore attack	Reference
Terpenoids Hemiterpenes	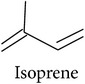		[16]
Monoterpenes	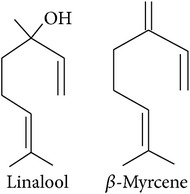	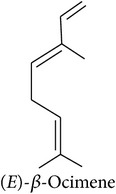	[16, 46, 83, 147]
Sesquiterpenes	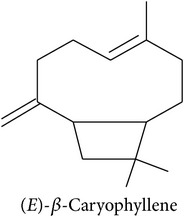	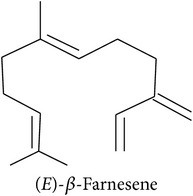	[46, 83, 147]

Benzenoids		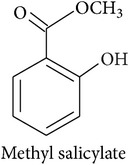	[16, 148]
Phenylpropanoids	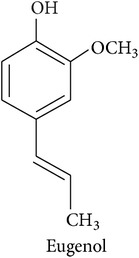		[20, 149]

Fatty acid derivatives		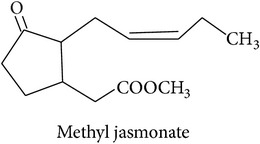	[20, 150]
“Green leaf volatiles”	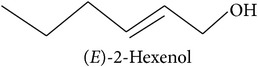	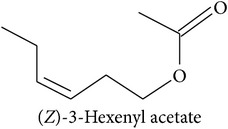	[46, 148]

**Table 3:** Degree of dietary specialization of several hymenoptera species regarding their insect host and their plant host as well as examples of insect and plant hosts.

Hymenoptera species	Specialist		Generalist		Plant species	Host species	References
	Regarding host plant	Regarding insect host	Regarding host plant	Regarding insect host			
*Aphelinus abdominalis*				X	Greenhouse crops, wheat	Cereal aphids (e.g., *Sitobion avenae*), greenhouse aphids (e.g.,* Myzus persicae *and* Macrosiphum euphorbiae*)	[103, 104]

*Aphidius colemani*				X	Wheat, cabbage, ascleipas, and others	Over 19 aphid species (e.g., *Aphis *spp*., Myzus persicae,* and* Rhopalosiphum padi*)	[105, 106]

*Aphidius funebris*				X		*Uroleucon *spp.	[107]

*Campoletis chlorideae*				X		Several noctuid caterpillar species (e.g., *Helicoverpa armigera*)	[108]

*Campoletis sonorensis*				X		Several noctuids caterpillar species	[61]

*Cotesia marginiventris*				X	Several, as the caterpillar species are polyphagous	Several caterpillar genera including *Heliothis *spp. and *Spodoptera *spp.	[89, 99]

*Cotesia sesamiae*				X		Wide range of stem borer species, mainly noctuid moth larvae (*Busseola fusca, Sesamia calamistis, Chilo orichalcocilielus, Chilo partellus*)	[109]

*Dacnusa sibirica*				X		*Liriomyza *spp. (e.g., *Liriomyza huidobrensis, L. bryoniae, *and *L. trifolii*)	[110]

*Diachasmimorpha longicaudata*				X		Tephritid fruit fly species (*Anastrepha, Ceratitis*,and* Bactrocera*)	[111, 112]

*Diadegma fenestrale*				X			[71]

*Lariophagus distinguendus*				X	Poaceae and Fabaceae	Over 11 different beetle species (e.g., *Rhyzopertha dominica* and* Sitophilus granarius*)	[113]

*Lysiphlebus testaceipes*				X	Bean, wheat, cabbage, and others	Over 9 aphid species (e.g.,* Aphis *spp*., Rhopalosiphum maidis* and* Toxoptera aurantii*)	[105, 114]

*Psyttalia concolor*				X			[101]

*Telenomus podisi*				X		Parasitizing the eggs of various pentatomids in agroecosystems (e.g., *Euchistus *spp., *Nezara viridula*, *Piezodorus guildinii*,and *P. maculiventris*)	[115, 116]

*Anaphes iole*			X	X	A variety of crop plants	*Lygus *spp.	[117]

*Aphidius ervi*			X	X	Wheat, cabbage, bean, and others	Over 14 aphid species (e.g.,* Acyrthosiphon pisum, Sitobion avenae,* and* Macrosiphum euphorbiae*)	[105, 118]

*Cotesia flavipes*			X	X	A variety of crops in the “New World”	Stem borers (e.g., *Diatraea saccharalis*, *D. grandiosella*, *Ostrinia nubilalis*, and *Chilo *spp.)	[119]

*Diaeretiella rapae*			X	X	Wheat, cabbage, bean, asclepias, and other crop plants	Over 23 aphid species (e.g., *Myzus persicae, Brevicoryne brassicae, *and *Sitobion avenae*)	[105, 120]

*Diglyphus isaea*			X	X	Mainly associated with herbaceous plants	18 different agromyzid species (*L. sativae*, e.g.)	[121]

*Opius dissitus*			X	X	Pea, celery (*L. huidobrensis*), vegetable leaf (*L. sativae*), and bean (*L. trifolii*)	*Liriomyza huidobrensis, L. sativae, L. trifolii*, and other leafminers	[122]

*Cotesia glomerata*		X		X		Pieridae spp. (e.g.,* P. brassicae, P. napi*,and* P. rapae*)	[97, 98, 123, 124]

*Apoanagyrus lopezi *(*= Epidinocarsis lopezi*)		X			*Manihot esculenta*	*Phenacoccus manihoti*	[125]

*Hyssopus pallidus*		X			Apple	*Cydia molesta* and *Cydia pomonella*	[124]

*Encarsia formosa*		X	X		Greenhouse crops like tomato and cucumber	*Trialeurodes vaporariorum* and* Bemisia tabaci* *Alternative host: aleurodes*	[126, 127]

*Trissolcus basalis*		X	X		Several	*Nezara viridula*	[128]

*Roptrocerus mirus*	X			X	Conifers	Bark beetle species	[129]

*Roptrocerus xylophagorum*	X			X	Conifers	Bark beetle species	[129, 130]

*Anagrus nilaparvatae*		X		X		*Laodelphax striatellus, Nilaparvata bakeri, Nilaparvata muiri, Nilaparvata lugens, Sogatella furcifera, Sogatella panicicola, Toya propinqua, *and *Toya tuberculosa*	[123, 131, 132]

*Aphidius rhopalosiphi*		X			Poaceae, for example, wheat	Aphids on Poaceae (e.g., *Metopolophium dirhodum, Rhopalosiphum padi, *and *Sitobion avenae*)	[133]

*Cardiochiles nigriceps*		X			Cotton, tobacco, and others	*Heliothis virescens*	[134]

*Chrysonotomyia ruforum*		X			*Pinus *spp.	Diprionidae of pine (e.g., *Diprion pini*)	[135]

*Cotesia kariyai*		X			Tobacco	*Pseudaletia separata*	[136]

*Cotesia plutellae*		X			Brassicaceae	*Plutella xylostella*	[137]

*Cotesia rubecula*		X				*Pieris rapae*	[98, 123]

*Diadegma semiclausum*		X			Brassicaceae	*Plutella xylostella*	[138, 139]

*Glyptapanteles flavicoxis*		X				*Lymantria dispar, L. obfuscata*	[140]

*Microplitis croceipes*		X			Over 100 species (*Heliothis *spp. is a generalist)	*Heliothis *spp.	[89, 99]

*Trybliographa rapae*		X			Cabbage	*Delia radicum*	[141]

*Oomyzus gallerucae*	X	X			*Ulmus *spp.	*Xanthogaleruca luteola*	[142]

*Orgilus lepidus*	X	X			Potato	*Phthorimaea operculella*	[143]

*Pauesia picta*	X	X			Scots pine (*Pinus sylvestris*)	*Cinara pinea*	[144, 145]

## References

[1] Peñuelas J., Llusià J. (2003). BVOCs: plant defense against climate warming?. *Trends in Plant Science*.

[2] Baldwin I. T. (2010). Plant volatiles. *Current Biology*.

[3] Holopainen J. K., Gershenzon J. (2010). Multiple stress factors and the emission of plant VOCs. *Trends in Plant Science*.

[4] Dicke M., Baldwin I. T. (2010). The evolutionary context for herbivore-induced plant volatiles: beyond the ‘cry for help’. *Trends in Plant Science*.

[5] Hilker M., Meiners T. (2010). How do plants ‘notice’ attack by herbivorous arthropods?. *Biological Reviews*.

[6] Holopainen J. K. (2004). Multiple functions of inducible plant volatiles. *Trends in Plant Science*.

[7] Rodriguez-Saona C., Kaplan I., Braasch J., Chinnasamy D., Williams L. (2011). Field responses of predaceous arthropods to methyl salicylate: a meta-analysis and case study in cranberries. *Biological Control*.

[8] Ode P. J., Wajnberg E., Colazza S. (2013). Plant defences and parasitoid chemical ecology. *Chemical Ecology of Insect Parasitoids*.

[9] Hare J. D. (2010). Ontogeny and season constrain the production of herbivore-inducible plant volatiles in the field. *Journal of Chemical Ecology*.

[10] Vet L. E. M., Dicke M. (1992). Ecology of infochemical use by natural enemies in a tritrophic context. *Annual Review of Entomology*.

[11] Yoneya K., Miki T. (2015). Co-evolution of foraging behaviour in herbivores and their natural enemies predicts multifunctionality of herbivore-induced plant volatiles. *Functional Ecology*.

[12] Gols R. (2014). Direct and indirect chemical defences against insects in a multitrophic framework: plant chemical defences against insects. *Plant, Cell & Environment*.

[13] Turlings T. C. J., Tumlinson J. H., Lewis W. J. (1990). Exploitation of herbivore-induced plant odors by host-seeking parasitic wasps. *Science*.

[14] Dicke M., Sabelis M. W., Takabayashi J. (1990). Do plants cry for help? Evidence related to a tritrophic system of predatory mites, spider mites and their host plants. *Insects-Plants '89*.

[15] Simpson M., Read D. M. Y., Gurr G. M., Wajnberg E., Colazza S. (2013). Application of chemical cues in arthropod pest management for organic crops. *Chemical Ecology of Insect Parasitoides*.

[16] Mumm R., Dicke M. (2010). Variation in natural plant products and the attraction of bodyguards involved in indirect plant defense. *Canadian Journal of Zoology*.

[17] Holopainen J. K., Himanen S. J., Poppy G. M., Wajnberg E., Colazza S. (2013). Climate change and its effects on the chemical ecology of insect parasitoids. *Chemical Ecology of Insect Parasitoides*.

[18] Schuman M. C., Barthel K., Baldwin I. T. (2012). Herbivory-induced volatiles function as defenses increasing fitness of the native plant *Nicotiana attenuata* in nature. *eLife*.

[19] Mithöfer A., Wanner G., Boland W. (2005). Effects of feeding Spodoptera littoralis on lima bean leaves. II. Continuous mechanical wounding resembling insect feeding is sufficient to elicit herbivory-related volatile emission. *Plant Physiology*.

[20] Dudareva N., Klempien A., Muhlemann J. K., Kaplan I. (2013). Biosynthesis, function and metabolic engineering of plant volatile organic compounds. *New Phytologist*.

[21] Venkatesan R. (2015). Biosynthesis and regulation of herbivore-induced plant volatile emission. *Journal of the Indian Institute of Science*.

[22] Dicke M. (2009). Behavioural and community ecology of plants that cry for help. *Plant, Cell & Environment*.

[23] Niinemets Ü., Kännaste A., Copolovici L. (2013). Quantitative patterns between plant volatile emissions induced by biotic stresses and the degree of damage. *Frontiers in Plant Science*.

[24] Peñuelas J., Staudt M. (2010). BVOCs and global change. *Trends in Plant Science*.

[25] Niinemets Ü., Loreto F., Reichstein M. (2004). Physiological and physicochemical controls on foliar volatile organic compound emissions. *Trends in Plant Science*.

[26] DeLucia E. H., Nabity P. D., Zavala J. A., Berenbaum M. R. (2012). Climate change: resetting plant-insect interactions. *Plant Physiology*.

[27] Sasaki K., Saito T., Lämsä M. (2007). Plants utilize isoprene emission as a thermotolerance mechanism. *Plant and Cell Physiology*.

[28] Siwko M. E., Marrink S. J., de Vries A. H., Kozubek A., Schoot Uiterkamp A. J. M., Mark A. E. (2007). Does isoprene protect plant membranes from thermal shock? A molecular dynamics study. *Biochimica et Biophysica Acta—Biomembranes*.

[29] Gouinguené S. P., Turlings T. C. J. (2002). The effects of abiotic factors on induced volatile emissions in corn plants. *Plant Physiology*.

[30] Lerdau M., Throop H. L. (2000). Sources of variability in isoprene emission and photosynthesis in two species of tropical wet forest trees. *Biotropica*.

[31] Peñuelas J., Munné-Bosch S. (2005). Isoprenoids: an evolutionary pool for photoprotection. *Trends in Plant Science*.

[32] Owen S. M., Peñuelas J. (2005). Opportunistic emissions of volatile isoprenoids. *Trends in Plant Science*.

[33] Vickers C. E., Gershenzon J., Lerdau M. T., Loreto F. (2009). A unified mechanism of action for volatile isoprenoids in plant abiotic stress. *Nature Chemical Biology*.

[34] Velikova V., Fares S., Loreto F. (2008). Isoprene and nitric oxide reduce damages in leaves exposed to oxidative stress. *Plant, Cell & Environment*.

[35] Himanen S. J., Nerg A.-M., Nissinen A. (2009). Effects of elevated carbon dioxide and ozone on volatile terpenoid emissions and multitrophic communication of transgenic insecticidal oilseed rape (*Brassica napus*). *New Phytologist*.

[36] Lavoir A. V., Staudt M., Schnitzler J. P. (2009). Drought reduced monoterpene emissions from *Quercus ilex* trees: results from a throughfall displacement experiment within a forest ecosystem. *Biogeosciences Discussions*.

[37] Guenther A. B., Zimmerman P. R., Harley P. C., Monson R. K., Fall R. (1993). Isoprene and monoterpene emission rate variability: model evaluations and sensitivity analyses. *Journal of Geophysical Research D*.

[38] Guenther A., Karl T., Harley P., Wiedinmyer C., Palmer P. I., Geron C. (2006). Estimates of global terrestrial isoprene emissions using MEGAN (Model of Emissions of Gases and Aerosols from Nature). *Atmospheric Chemistry and Physics*.

[39] Lavoir A. V., Duffet C., Mouillot F. (2011). Scaling-up leaf monoterpene emissions from a water limited *Quercus ilex* woodland. *Atmospheric Environment*.

[40] Ormeño E., Fernandez C., Bousquet-Mélou A. (2007). Monoterpene and sesquiterpene emissions of three Mediterranean species through calcareous and siliceous soils in natural conditions. *Atmospheric Environment*.

[41] Rivoal A., Fernandez C., Lavoir A.-V. (2010). Environmental control of terpene emissions from *Cistus monspeliensis* L. in natural Mediterranean shrublands. *Chemosphere*.

[42] Fares S., Brilli F., Noguès I. (2008). Isoprene emission and primary metabolism in *Phragmites australis* grown under different phosphorus levels: isoprene emission and primary metabolism. *Plant Biology*.

[43] Teuber M., Zimmer I., Kreuzwieser J. (2008). VOC emissions of Grey poplar leaves as affected by salt stress and different N sources: VOC emissions and salt stress in poplar. *Plant Biology*.

[44] Loreto F., Delfine S. (2000). Emission of isoprene from salt-stressed *Eucalyptus globulus* leaves. *Plant Physiology*.

[45] Rhoades D. F. (1979). Evolution of plant chemical defense against herbivores. *Herbivores: Their Interaction with Secondary Plant Metabolites*.

[46] Robert C. A. M., Erb M., Hiltpold I. (2013). Genetically engineered maize plants reveal distinct costs and benefits of constitutive volatile emissions in the field. *Plant Biotechnology Journal*.

[47] Kessler A. (2015). The information landscape of plant constitutive and induced secondary metabolite production. *Current Opinion in Insect Science*.

[48] Arimura G.-I., Kost C., Boland W. (2005). Herbivore-induced, indirect plant defences. *Biochimica et Biophysica Acta—Molecular and Cell Biology of Lipids*.

[49] Han P., Lavoir A.-V., Le Bot J., Amiens-Desneux E., Desneux N. (2014). Nitrogen and water availability to tomato plants triggers bottom-up effects on the leafminer *Tuta absoluta*. *Scientific Reports*.

[50] Seidl-Adams I., Richter A., Boomer K. B., Yoshinaga N., Degenhardt J., Tumlinson J. H. (2014). Emission of herbivore elicitor-induced sesquiterpenes is regulated by stomatal aperture in maize (*Zea mays*) seedlings: guard cells regulate sesquiterpene emission. *Plant, Cell & Environment*.

[51] Cai X.-M., Sun X.-L., Dong W.-X., Wang G.-C., Chen Z.-M. (2014). Herbivore species, infestation time, and herbivore density affect induced volatiles in tea plants. *Chemoecology*.

[52] Loughrin J. H., Manukian A., Heath R. R., Turlings T. C. J., Tumlinson J. H. (1994). Diurnal cycle of emission of induced volatile terpenoids by herbivore-injured cotton plant. *Proceedings of the National Academy of Sciences of the United States of America*.

[53] Harley P., Deem G., Flint S., Caldwell M. (1996). Effects of growth under elevated UV-B on photosynthesis and isoprene emission in *Quercus gambelii* and *Mucuna pruriens*. *Global Change Biology*.

[54] Ibrahim M. A., Stewart-Jones A., Pulkkinen J., Poppy G. M., Holopainen J. K. (2008). The influence of different nutrient levels on insect-induced plant volatiles in Bt and conventional oilseed rape plants: oilseed rape emissions and soil nutrient levels. *Plant Biology*.

[55] Lou Y., Baldwin I. T. (2004). Nitrogen supply influences herbivore-induced direct and indirect defenses and transcriptional responses in *Nicotiana attenuata*. *Plant Physiology*.

[56] Winter T. R., Rostás M. (2010). Nitrogen deficiency affects bottom-up cascade without disrupting indirect plant defense. *Journal of Chemical Ecology*.

[57] Forieri I., Hildebrandt U., Rostás M. (2016). Salinity stress effects on direct and indirect defence metabolites in maize. *Environmental and Experimental Botany*.

[58] Winter T. R., Borkowski L., Zeier J., Rostás M. (2012). Heavy metal stress can prime for herbivore-induced plant volatile emission: copper primes VOCs. *Plant, Cell & Environment*.

[59] Stam J. M., Kroes A., Li Y. (2014). Plant interactions with multiple insect herbivores: from community to genes. *Annual Review of Plant Biology*.

[60] Stiling P., Cornelissen T. (2007). How does elevated carbon dioxide (CO_2_) affect plant-herbivore interactions? A field experiment and meta-analysis of CO_2_-mediated changes on plant chemistry and herbivore performance. *Global Change Biology*.

[61] Gouinguené S., Pickett J. A., Wadhams L. J., Birkett M. A., Turlings T. C. J. (2005). Antennal electrophysiological responses of three parasitic wasps to caterpillar-induced volatiles from maize (*Zea mays mays*), cotton (*Gossypium herbaceum*), and cowpea (*Vigna unguiculata*). *Journal of Chemical Ecology*.

[62] Caputo C., Rutitzky M., Ballaré C. L. (2006). Solar ultraviolet-B radiation alters the attractiveness of Arabidopsis plants to diamondback moths (*Plutella xylostella* L.): impacts on oviposition and involvement of the jasmonic acid pathway. *Oecologia*.

[63] Foggo A., Higgins S., Wargent J. J., Coleman R. A. (2007). Tri-trophic consequences of UV-B exposure: plants, herbivores and parasitoids. *Oecologia*.

[64] Winter T. R., Rostás M. (2008). Ambient ultraviolet radiation induces protective responses in soybean but does not attenuate indirect defense. *Environmental Pollution*.

[65] Vuorinen T., Nerg A.-M., Ibrahim M. A., Reddy G. V. P., Holopainen J. K. (2004). Emission of *Plutella xylostella*-induced compounds from cabbages grown at elevated CO_2_ and orientation behavior of the natural enemies. *Plant Physiology*.

[66] Pinto D. M., Blande J. D., Nykänen R., Dong W.-X., Nerg A.-M., Holopainen J. K. (2007). Ozone degrades common herbivore-induced plant volatiles: does this affect herbivore prey location by predators and parasitoids?. *Journal of Chemical Ecology*.

[67] Loivamäki M., Mumm R., Dicke M., Schnitzler J.-P. (2008). Isoprene interferes with the attraction of bodyguards by herbaceous plants. *Proceedings of the National Academy of Sciences of the United States of America*.

[68] Schmelz E. A., Alborn H. T., Engelberth J., Tumlinson J. H. (2003). Nitrogen deficiency increases volicitin-induced volatile emission, jasmonic acid accumulation, and ethylene sensitivity in maize. *Plant Physiology*.

[69] Adler L. S., Wink M., Distl M., Lentz A. J. (2006). Leaf herbivory and nutrients increase nectar alkaloids. *Ecology Letters*.

[70] Desneux N., Barta R. J., Hoelmer K. A., Hopper K. R., Heimpel G. E. (2009). Multifaceted determinants of host specificity in an aphid parasitoid. *Oecologia*.

[71] Gols R., Veenemans C., Potting R. P. J. (2012). Variation in the specificity of plant volatiles and their use by a specialist and a generalist parasitoid. *Animal Behaviour*.

[72] Kessler A., Halitschke R., Poveda K. (2011). Herbivory-mediated pollinator limitation: negative impacts of induced volatiles on plant-pollinator interactions. *Ecology*.

[73] Schiestl F. P., Kirk H., Bigler L., Cozzolino S., Desurmont G. A. (2014). Herbivory and floral signaling: phenotypic plasticity and tradeoffs between reproduction and indirect defense. *New Phytologist*.

[74] Halitschke R., Stenberg J. A., Kessler D., Kessler A., Baldwin I. T. (2008). Shared signals—‘alarm calls’ from plants increase apparency to herbivores and their enemies in nature. *Ecology Letters*.

[75] Thomson L. J., Macfadyen S., Hoffmann A. A. (2010). Predicting the effects of climate change on natural enemies of agricultural pests. *Biological Control*.

[76] Mathur V., Tytgat T. O. G., Hordijk C. A. (2013). An ecogenomic analysis of herbivore-induced plant volatiles in *Brassica juncea*. *Molecular Ecology*.

[77] James D. G., Grasswitz T. R. (2005). Synthetic herbivore-induced plant volatiles increase field captures of parasitic wasps. *Biocontrol*.

[78] Kaplan I. (2012). Attracting carnivorous arthropods with plant volatiles: the future of biocontrol or playing with fire?. *Biological Control*.

[79] Rodriguez-Saona C., Blaauw B. R., Isaacs R., Rodriguez-Saona C. (2012). Manipulation of natural enemies in agroecosystems: habitat and semiochemicals for sustainable insect pest control. *Integrated Pest Management and Pest Control Current and Future Tactics*.

[80] Braasch J., Wimp G. M., Kaplan I. (2012). Testing for phytochemical synergism: arthropod community responses to induced plant volatile blends across crops. *Journal of Chemical Ecology*.

[81] Gols R., Bullock J. M., Dicke M., Bukovinszky T., Harvey J. A. (2011). Smelling the wood from the trees: non-linear parasitoid responses to volatile attractants produced by wild and cultivated cabbage. *Journal of Chemical Ecology*.

[82] Tamiru A., Bruce T., Woodcock C. (2015). Chemical cues modulating electrophysiological and behavioural responses in the parasitic wasp *Cotesia sesamiae*. *Canadian Journal of Zoology*.

[83] Fontana A., Held M., Fantaye C. A., Turlings T. C., Degenhardt J., Gershenzon J. (2011). Attractiveness of constitutive and herbivore-induced sesquiterpene blends of maize to the parasitic wasp *Cotesia marginiventris* (cresson). *Journal of Chemical Ecology*.

[84] Maeda T., Kishimoto H., Wright L. C., James D. G. (2015). Mixture of synthetic herbivore-induced plant volatiles attracts more *Stethorus punctum picipes* (Casey) (Coleoptera: Coccinellidae) than a single volatile. *Journal of Insect Behavior*.

[85] van Wijk M., de Bruijn P. J. A., Sabelis M. W. (2011). Complex odor from plants under attack: herbivore's enemies react to the whole, not its parts. *PLoS ONE*.

[86] Braasch J., Kaplan I. (2012). Over what distance are plant volatiles bioactive? Estimating the spatial dimensions of attraction in an arthropod assemblage. *Entomologia Experimentalis et Applicata*.

[87] Meiners T., Peri E., Wajnberg E., Colazza S. (2013). Chemical ecology of insect parasitoids: essential elements for developing effective biological control programmes. *Chemical Ecology of Insect Parasitoids*.

[88] Orre G. U. S., Wratten S. D., Jonsson M., Hale R. J. (2010). Effects of an herbivore-induced plant volatile on arthropods from three trophic levels in brassicas. *Biological Control*.

[89] Ngumbi E., Fadamiro H. (2012). Species and sexual differences in behavioural responses of a specialist and generalist parasitoid species to host-related volatiles. *Bulletin of Entomological Research*.

[90] Ngumbi E., Chen L., Fadamiro H. (2010). Electroantennogram (EAG) responses of *Microplitis croceipes* and *Cotesia marginiventris* and their lepidopteran hosts to a wide array of odor stimuli: correlation between EAG response and degree of host specificity?. *Journal of Insect Physiology*.

[91] Ngumbi E., Chen L., Fadamiro H. Y. (2009). Comparative GC-ead responses of a specialist (*Microplitis croceipes*) and a generalist (*Cotesia marginiventris*) parasitoid to cotton volatiles induced by two caterpillar species. *Journal of Chemical Ecology*.

[92] Cortesero A. M., De Moraes C. M., Stapel J. O., Tumlinson J. H., Lewis W. J. (1997). Comparisons and contrasts in host-foraging strategies of two larval parasitoids with different degrees of host specificity. *Journal of Chemical Ecology*.

[93] Smid H. M., van Loon J. J. A., Posthumus M. A., Vet L. E. M. (2002). GC-EAG-analysis of volatiles from Brussels sprouts plants damaged by two species of *Pieris* caterpillars: olfactory receptive range of a specialist and a generalist parasitoid wasp species. *Chemoecology*.

[94] Smid H. M., Dicke M., Takken W. (2006). Variation in learning of herbivory-induced plant odours by parasitic wasps: from brain to behaviour. *Chemical Ecology: From Gene to Ecosystem*.

[95] Lewis W. J., Tumlinson J. H. (1988). Host detection by chemically mediated associative learning in a parasitic wasp. *Nature*.

[96] Lewis W. J., Takasu K. (1990). Use of learned odours by a parasitic wasp in accordance with host and food needs. *Nature*.

[97] Lucas-Barbosa D., Poelman E. H., Aartsma Y., Snoeren T. A. L., van Loon J. J. A., Dicke M. (2014). Caught between parasitoids and predators—survival of a specialist herbivore on leaves and flowers of mustard plants. *Journal of Chemical Ecology*.

[98] Vos M., Hemerik L., Vet L. E. M. (1998). Patch exploitation by the parasitoids *Cotesia rubecula* and *Cotesia glomerata* in multi-patch environments with different host distributions. *Journal of Animal Ecology*.

[99] Ngumbi E., Jordan M., Fadamiro H. (2012). Comparison of associative learning of host-related plant volatiles in two parasitoids with different degrees of host specificity, *Cotesia marginiventris* and *Microplitis croceipes*. *Chemoecology*.

[100] Steidle J. L. M., van Loon J. J. A. (2003). Dietary specialization and infochemical use in carnivorous arthropods: testing a concept. *Entomologia Experimentalis et Applicata*.

[101] Canale A., Geri S., Benelli G. (2014). Associative learning for host-induced fruit volatiles in *Psyttalia concolor* (Hymenoptera: Braconidae), a koinobiont parasitoid of tephritid flies. *Bulletin of Entomological Research*.

[102] Benelli G., Stefanini C., Giunti G., Geri S., Messing R. H., Canale A. (2014). Associative learning for danger avoidance nullifies innate positive chemotaxis to host olfactory stimuli in a parasitic wasp. *Naturwissenschaften*.

[103] Enkegaard A., Sigsgaard L., Kristensen K. (2013). Shallot Aphids, *Myzus ascalonicus*, in strawberry: biocontrol potential of three predators and three parasitoids. *Journal of Insect Science*.

[104] Mölck G., Pinn H., Wyss U. (2000). Manipulation of plant odour preference by learning in the aphid parasitoid *Aphelinus abdominalis* (Hymenoptera: Aphelinidae). *European Journal of Entomology*.

[105] Kavallieratos N. G., Tomanović Ž., Starý P. (2004). A survey of aphid parasitoids (Hymenoptera: Braconidae: Aphidiinae) of Southeastern Europe and their aphid-plant associations. *Applied Entomology and Zoology*.

[106] Storeck A., Poppy G. M., Emden H. F., Powell W. (2000). The role of plant chemical cues in determining host preference in the generalist aphid parasitoid *Aphidius colemani*. *Entomologia Experimentalis et Applicata*.

[107] Völkl W., Kranz P., Weisser W., Hübner G. (1995). Patch time allocation and resource exploitation in aphid primary parasitoids and hyperparasitoids searching simultaneously within aphid colonies. *Journal of Applied Entomology*.

[108] Yan Z.-G., Wang C.-Z. (2006). Similar attractiveness of maize volatiles induced by *Helicoverpa armigera* and *Pseudaletia separata* to the generalist parasitoid *Campoletis chlorideae*. *Entomologia Experimentalis et Applicata*.

[109] Branca A., Le Ru B. P., Vavre F., Silvain J.-F., Dupas S. (2011). Intraspecific specialization of the generalist parasitoid *Cotesia sesamiae* revealed by polyDNAvirus polymorphism and associated with different *Wolbachia* infection. *Molecular Ecology*.

[110] Abe Y., Takeuchi T., Tokumaru S., Kamata J. (2005). Comparison of the suitability of three pest leafminers (Diptera: Agromyzidae) as hosts for the parasitoid *Dacnusa sibirica* (Hymenoptera: Braconidae). *European Journal of Entomology*.

[111] Cicero L., Sivinski J., Aluja M. (2012). Effect of host diet and adult parasitoid diet on egg load dynamics and egg size of braconid parasitoids attacking *Anastrepha ludens*. *Physiological Entomology*.

[112] Julsirikul D., Worapong J., Kitthawee S. (2014). Analysis of mitochondrial *COI* sequences of the *Diachasmimorpha longicaudata* (Hymenoptera: Braconidae) species complex in Thailand: COI sequences of *D. longicaudata*. *Entomological Science*.

[113] Steidle J. L. M., Steppuhn A., Ruther J. (2003). Specific foraging kairomones used by a generalist parasitoid. *Journal of Chemical Ecology*.

[114] Grasswitz T. R., Paine T. D. (1993). Effect of experience on in-flight orientation to host-associated cues in the generalist parasitoid *Lysiphlebus testaceipes*. *Entomologia Experimentalis et Applicata*.

[115] Bruni R., Sant'Ana J., Aldrich J. R., Bin F. (2000). Influence of host pheromone on egg parasitism by Scelionid wasps: comparison of phoretic and nonphoretic parasitoids. *Journal of Insect Behavior*.

[116] Laumann R. A., Aquino M. F. S., Moraes M. C. B., Pareja M., Borges M. (2009). Response of the egg parasitoids *Trissolcus basalis* and *Telenomus podisi* to compounds from defensive secretions of stink bugs. *Journal of Chemical Ecology*.

[117] Manrique V., Jones W. A., Williams L. H., Bernal J. S. (2005). Olfactory responses of *Anaphes iole* (Hymenoptera: Mymaridae) to volatile signals derived from host habitats. *Journal of Insect Behavior*.

[118] Henry L. M., Gillespie D. R., Roitberg B. D. (2005). Does mother really know best? Oviposition preference reduces reproductive performance in the generalist parasitoid *Aphidius ervi*. *Entomologia Experimentalis et Applicata*.

[119] Mahmoud A. M. A., De Luna-Santillana E. J., Guo X., Reyes-Villanueva F., Rodríguez-Pérez M. A. (2012). Development of the braconid wasp *Cotesia flavipes* in two Crambids, *Diatraea saccharalis* and Eoreuma loftini: evidence of host developmental disruption. *Journal of Asia-Pacific Entomology*.

[120] Antolin M. F., Bjorksten T. A., Vaughn T. T. (2006). Host-related fitness trade-offs in a presumed generalist parasitoid, *Diaeretiella rapae* (Hymenoptera: Aphidiidae). *Ecological Entomology*.

[121] Zhao Y. X., Kang L. (2002). The role of plant odours in the leafminer *Liriomyza sativae* (Diptera: Agromyzidae) and its parasitoid *Diglyphus isaea* (Hymenoptera: Eulophidae): orientation towards the host habitat. *European Journal of Entomology*.

[122] http://entnemdept.ufl.edu/creatures/beneficial/wasps/opius_dissitus.htm.

[123] Brodeur J., Geervliet J. B. F., Vet L. E. M. (1996). The role of host species, age and defensive behaviour on ovipositional decisions in a solitary specialist and gregarious generalist parasitoid (*Cotesia* species). *Entomologia Experimentalis et Applicata*.

[124] Collatz J., Dorn S. (2013). Tritrophic consequences arising from a host shift between apple and walnut in an oligophagous herbivore. *Biological Control*.

[125] Souissi R., Rü B. (1999). Behavioural responses of the endoparasitoid *Apoanagyrus lopezi* to odours of the host and host's cassava plants. *Entomologia Experimentalis et Applicata*.

[126] Guerrieri E. (1997). Flight behaviour of *Encarsia formosa* in response to plant and host stimuli. *Entomologia Experimentalis et Applicata*.

[127] Polaszek A., Evans G. A., Bennett F. D. (1992). *Encarsia parasitoids* of *Bemisia tabaci* (Hymenoptera: Aphelinidae, Homoptera: Aleyrodidae): a preliminary guide to identification. *Bulletin of Entomological Research*.

[128] Salerno G., Conti E., Peri E., Colazza S., Bin F. (2006). Kairomone involvement in the host specificity of the egg parasitoid *Trissolcus basalis* (Hymenoptera: Scelionidae). *European Journal of Entomology*.

[129] Pettersson E. M. (2001). Volatile attractants for three Pteromalid parasitoids attacking concealed spruce bark beetles. *Chemoecology*.

[130] Samson P. R. (1984). The biology of *Roptrocerus xylophagorum* [Hym.: Torymidae], with a note on its taxonomic status. *Entomophaga*.

[131] Gurr G. M., Liu J., Read D. M. Y. (2011). Parasitoids of Asian rice planthopper (Hemiptera: Delphacidae) pests and prospects for enhancing biological control by ecological engineering. *Annals of Applied Biology*.

[132] Xu H., He X., Zheng X., Yang Y., Tian J., Lu Z. (2014). Infection of rice plants by rice black streaked dwarf virus improves an egg parasitoid, *Anagrus nilaparvatae* (Hymenoptera: Mymaridae), of rice planthoppers. *Environmental Entomology*.

[133] Stilmant D., Van Bellinghen C., Hance T., Boivin G. (2008). Host specialization in habitat specialists and generalists. *Oecologia*.

[134] De Moraes C. M., Lewis W. J., Pare P. W., Alborn H. T., Tumiinson J. H. (1998). Herbivore-infested plants selectively attract parasitoids. *Nature*.

[135] Mumm R., Tiemann T., Varama M., Hilker M. (2005). Choosy egg parasitoids: specificity of oviposition-induced pine volatiles exploited by an egg parasitoid of pine sawflies. *Entomologia Experimentalis et Applicata*.

[136] Mandour N. S., Kainoh Y., Ozawa R., Uefune M., Takabayashi J. (2013). Effects of prohydrojasmon-treated corn plants on attractiveness to parasitoids and the performance of their hosts: effects of PDJ-treatment on corn plants. *Journal of Applied Entomology*.

[137] Roux O., Gers C., Tene-Ghomsi J. N., Arvanitakis L., Bordat D., Legal L. (2007). Chemical characterization of contact semiochemicals for host-recognition and host-acceptance by the specialist parasitoid *Cotesia plutellae* (Kurdjumov). *Chemoecology*.

[138] Bruinsma M., Posthumus M. A., Mumm R., Mueller M. J., Van Loon J. J. A., Dicke M. (2009). Jasmonic acid-induced volatiles of *Brassica oleracea* attract parasitoids: effects of time and dose, and comparison with induction by herbivores. *Journal of Experimental Botany*.

[139] Roßbach A., Löhr B., Vidal S. (2006). Does a specialist parasitoid adapt to its host on a new host plant?. *Journal of Insect Behavior*.

[140] Fuester R. W., Swan K. S., Taylor P. B., Ramaseshiah G. (2008). Effects of parent age at mating on reproductive response of *Glyptapanteles flavicoxis* (Hymenoptera: Braconidae), a larval parasitoid of the gypsy moth (Lepidoptera: Lymantriidae). *Journal of Economic Entomology*.

[141] Pierre P. S., Jansen J. J., Hordijk C. A., van Dam N. M., Cortesero A.-M., Dugravot S. (2011). Differences in volatile profiles of turnip plants subjected to single and dual herbivory above- and belowground. *Journal of Chemical Ecology*.

[142] Meiners T., Westerhaus C., Hilker M. (2000). Specificity of chemical cues used by a specialist egg parasitoid during host location. *Entomologia Experimentalis et Applicata*.

[143] Keller M. A., Horne P. A. (1993). Sources of host-location cues for the parasitic wasp *Orgilus lepidus* (Braconidae). *Australian Journal of Zoology*.

[144] Völkl W. (2000). Foraging behaviour and sequential multisensory orientation in the aphid parasitoid, *Pauesia picta* (Hym., Aphidiidae) at different spatial scales. *Journal of Applied Entomology*.

[145] Völkl W. (2001). Parasitoid learning during interactions with ants: how to deal with an aggressive antagonist. *Behavioral Ecology and Sociobiology*.

[146] Gigot C., Ongena M., Fauconnier M.-L., Wathelet J.-P., du Jardin P., Thonart P. (2010). The lipoxygenase metabolic pathway in plants: potential for industrial production of natural green leaf volatiles. *Biotechnology, Agronomy and Society and Environment*.

[147] Unsicker S. B., Kunert G., Gershenzon J. (2009). Protective perfumes: the role of vegetative volatiles in plant defense against herbivores. *Current Opinion in Plant Biology*.

[148] Dickens J. C. (1999). Predator-prey interactions: olfactory adaptations of generalist and specialist predators. *Agricultural and Forest Entomology*.

[149] Gang D. R., Wang J., Dudareva N. (2001). An investigation of the storage and biosynthesis of phenylpropenes in sweet basil. *Plant Physiology*.

[150] Farmer E. E., Ryan C. A. (1990). Interplant communication: airborne methyl jasmonate induces synthesis of proteinase inhibitors in plant leaves. *Proceedings of the National Academy of Sciences of the United States of America*.

